# Guidance on defining the scope and development of text-based coaching protocols for digital mental health interventions

**DOI:** 10.1177/2055207619896145

**Published:** 2019-12-16

**Authors:** Emily G Lattie, Andrea K Graham, Heather D Hadjistavropoulos, Blake F Dear, Nickolai Titov, David C Mohr

**Affiliations:** 1 Center for Behavioral Intervention Technologies, Northwestern University, Chicago, IL, USA; 2 Department of Psychology, University of Regina, Regina, SK, Canada; 3 Department of Psychology, Macquarie University, Sydney, NSW, Australia

**Keywords:** Digital health, behavior change, coaching, engagement

## Abstract

A body of literature suggests that the provision of human support improves both adherence to and clinical outcomes for digital mental health interventions. While multiple models of providing human support, or coaching, to support digital mental health interventions have been introduced, specific guidance on how to develop coaching protocols has been lacking. In this Education Piece, we provide guidance on developing coaching protocols for text-based communication in digital mental health interventions. Researchers and practitioners who are tasked with developing coaching protocols are prompted to consider the scope of coaching for the intervention, the selection and training of coaches, specific coaching techniques, how to structure communication with clients and how to monitor adherence to guidelines, and quality of coaching. Our goal is to advance thinking about the provision of human support in digital mental health interventions to inform stronger, more engaging, and effective intervention designs.

In the last two decades, there has been a rapid proliferation of digital mental health interventions. Digital mental health interventions (i.e. delivered via app-based and web-based platforms) offer the potential to greatly expand accessibility to behavioral interventions, which are often viewed by clients as preferable to medication, but difficult to access.^1,2^ A body of literature has demonstrated the benefits of human support for reinforcing adherence to digital mental health interventions and improving clinical outcomes.^3–5^ These types of programs, offered with human support, are often referred to as guided self-help. Different models of support have been developed including the Swedish Experience,^6^ the Macquarie University Model,^7^ Supportive Accountability,^8^ and the Efficiency Model.^9^

While these models offer theoretical and conceptual guidance, there is a notable lack of practical guidance in the published literature on how to define and develop a support protocol for digital mental health interventions. The goal of this paper is to offer guidance on defining and developing support protocols for digital mental health interventions targeting individuals. While some of this guidance may be applicable to providing support in digital mental health interventions targeting couples or families, here we focus primarily on supported individual-level interventions in both research and clinical practice settings. A variety of terms have been used to describe the person who provides support, including therapist, clinician, guide, supporter, and coach. In this paper we use the term coach, as it does not imply a particular level or type of training. We contend that variables that need to be considered when developing guidelines for offering support to accompany digital mental health interventions are similar, regardless of training.

Here, we present primarily on text-based coaching (e.g. Short Messaging Service (SMS) and messages sent through email-type services) because studies have found text-based coaching yields comparable outcomes to telephone-based coaching^10,11^ and text-based coaching is increasingly used as a primary method of communication.^12^ Of note, text-based coaching often includes some telephone-based coaching, and telephone-based coaching often includes some text-based coaching. In general, text-based coaching uses less coach time and has added convenience in terms of not requiring scheduled appointments. While a thorough discussion of telephone-based coaching is beyond the scope of this review, we briefly comment on how the suggestions presented here for text-based coaching may be relevant for the development of telephone-based coaching guidelines. We also offer considerations to delineate telephone-based coaching for a digital health intervention from telephone-based counseling or therapy.

## Defining the scope of text-based coaching

To provide some framing for a text-based coaching protocol, we first highlight a few overarching features of text-based coaching that are relevant for guiding subsequent decisions about the development of a coaching protocol. First, coaching is not the same as delivering psychotherapy. In psychotherapy, the clinician is responsible for introducing and supporting skill acquisition. While structured materials are used in psychotherapy, the clinician typically presents information verbally, which is then comingled with support, emotional exploration, and guided discovery. In contrast, guided digital mental health interventions most commonly attempt to separate these two functions. The initial presentation of psychoeducation and skills typically is provided by the digital tools, while the human coach subsequently provides support and assistance with the acquisition and application of knowledge and skills. The nature of support in coaching can also vary from primarily being focused on encouraging intervention usage to being more collaborative in nature, focused on guiding the acquisition of insight, knowledge, skills, or solving problems in the client’s life.

A text-based coaching protocol requires three broad categories of definitions: the workflow, the timing, and the content of messages.

### Coaching workflow

Coaching digital mental health interventions presents a far different workflow than traditional face-to-face therapies. While a therapist may have the structure of routinely scheduled 45–60-minute sessions with clients, a coach is unlikely to have the once-a-week 45–60-minute structure with program clients. Coaching protocols typically define the timing and content of outreach messages to the clients; however, to the extent clients engage in a text-based dialogue, coaches sometimes communicate with clients outside prescribed outreach points. Prescribed coach outreach can vary considerably. Many existing protocols use weekly 10–15-minute outreach;^13–16^ however, this can vary from no outreach (coaches are available but there is little to no outreach^17,18^) to contacts multiple times per week.^19^ To prepare an appropriate protocol, it is important to consider the workflow and availability of coaches, including coaching-related tasks and any other duties related to the position, such as intake interviews or collection of follow-up questionnaires to assess outcome.

### Coaching timing

The timing of coaching is unique for text-based interventions, as the asynchronous nature of text-based coaching is different from face-to-face or telephone-based coaching, and has benefits and drawbacks. One benefit of asynchronicity is the opportunity to craft messages in a more targeted way than might be possible in a face-to-face conversation, which offers the benefit that coaches have the capacity to say exactly what they want to say to clients. However, the timing of a response may cause interpretation problems, in that a delayed response may be purposeful (e.g. if a client is upset or frustrated by a message from a coach, or a client perceives that a delayed response from a coach is because of something they said) or merely the result of differing schedules. Indeed, although the principles of asynchronous communication are consistent with how individuals engage in text messaging in their daily routines, different people use communication technologies in different ways – some view SMS-based messages as more urgent than telephone calls and expect them to be responded to almost immediately, while others view SMS-based messages as a much less urgent form of communication. Clients may have different expectations regarding their interactions with a coach within the context of a health-focused intervention. The standards around response latency between coach and client is something that should be addressed early in a coaching relationship. In digital mental health interventions, it is strongly recommended to set expectations at the onset of the relationship, such as “*Whenever I get a message from you, I will get back to you within one business day. If you can do the same it will help keep our communication going*.” Additionally, if a coaching protocol intends to limit coaching services to business hours only, boundaries should be outlined regarding the hours for communication and the timing by which coaches may be expected to respond to clients’ messages. An important consideration is whether automated emails will be a part of the digital mental health intervention and what nature these emails will take (e.g. reminder only or additional educational information and encouragement). Automated emails are known to decrease burden on coaches and also improve adherence and clinical outcomes in those with elevated symptoms.^20^

Particularly in the event of an emergency or crisis, clients should not anticipate immediate responses from the coach, if the capacity to respond immediately is not available. Teams developing or implementing a digital intervention might consider different ways to address or offset this potential type of outreach from clients (e.g. offering an auto-response for crisis-related messages, making a list of resources or safety information accessible to clients somewhere in the intervention, encouraging clients to develop safety plans^21^). Differentiating both of these points is important for coaches and intervention clients, and we encourage a coaching protocol to clearly delineate these concepts in client-facing materials.

### Coaching content

Regarding the content of coaching messages, there is an issue with the narrow bandwidth available. Bite-size pieces of information lend greater power to the words, and thus require more deliberate crafting. Some successful coaching protocols use highly templated messages, in which coaches can modify, personalize, and add information as relevant to the progress of the specific client. Having such message templates available to coaches can facilitate a more efficient coach workflow once coaches have become familiarized with the template options, and can help coaches strike an appropriate conversational tone (i.e. not too formal and not too casual). A helpful method for creating templates is to conduct a qualitative analysis of common client questions in emails.^22^ These can then be used to formulate templated responses to the common questions. Of note, while many client questions are related to program content, other questions concern technical challenges, the nature of the coaching relationship, or questions that are outside the program area.

It is also important to note that text-based conversation lacks non-verbal cues and the nuances of tone. With regard to content, coaches need to be attentive to how messages are crafted to ensure the points of conversation are clear, particularly those that may be subject to interpretation. Because social interpretation may be difficult to control, there is a greater risk in using humor and, in general, may be best to avoid. Emoticons also have the potential to be misunderstood. We generally recommend using these sparingly and only when clients use emoticons themselves.

With this scope of work in mind, we now turn to the key considerations in developing a coaching protocol.

## Coaches

### Coach characteristics

One of the first considerations for developing a coaching protocol is: who will be the coaches? Two important issues to consider are the level of mental health training and amount of time available. While many Internet-based cognitive behavioral therapy (iCBT) programs have used staff with clinical degrees,^23,24^ there is evidence of efficacy for using lay- and bachelor’s-trained individuals in digital mental health interventions.^25,26^ Nevertheless, it should be noted that when lay- and bachelor’s-trained individuals have been used in research studies, considerable training, structure, and supervision have been made available and, thus, this should be considered when using coaches who are not trained mental health professionals. As previously described, coaching is not psychotherapy,^27^ and when the role of the coach is focused on helping clients engage with the intervention and support the acquisition of skills addressed in the intervention, the use of lay- or bachelor-level coaches may be appropriate, with the proper training. Consideration of both the background of coaches and their other work responsibilities will make developing a coaching protocol (and training on the protocol) go more smoothly. For example, the coaching protocol for a post-baccalaureate research assistant would be different from the coaching protocol for a psychologist or social worker. Furthermore, the protocol will also need to differ if individuals work full-time compared to part-time while maintaining other existing job responsibilities. Some past research suggests that those who provide coaching while managing other responsibilities have lower adherence to pre-specified guidelines for coaching.^28^

### Coach training

Training of coaches may focus on both the specific and non-specific components of delivering the coaching protocol. Coaches need to develop an understanding of the specific problems of the population, and an understanding of the specific intervention including its goals and its potential shortfalls. This is similar to guidelines for traditional health coaching (i.e. health coaching provided in person or by telephone, without an app-based or web-based program^27^); a basic understanding of the intervention’s targeted problem area is key, as it forms the basis for the types of skills that the coach is supporting clients in acquiring. As such, training in the coaching protocol may need to include training in the basic content area of the intervention to ensure the coach is a credible and effective supporter.^29^ For example, it would be helpful for coaches delivering an intervention focused on depression to recognize that amotivation is a key symptom of the disorder, which might manifest during the coaching period as difficulties engaging with the intervention. By having this understanding of the symptoms of depression, coaches will be able to offer validation and encouragement that is appropriately tailored to the intervention and client. Non-specific factors, such as how to establish a bond or an alliance with a client, may also need to be a part of coach training, particularly for coaches coming from non-clinical backgrounds or for those who are new to digital interventions.

To promote appropriate skill use, coaches also need to be trained to know the intervention well. Our experience shows that coaches are most effective with clients when they have thorough knowledge of the clinical targets of the intervention, and how and when these clinical targets are delivered via the technology. Indeed, coaches who lack this understanding of the intervention can be challenged to sufficiently address clients’ questions and comments about their progress. It can be beneficial for coach training to include having coaches practice using the intervention for a structured period of time to gain a client’s perspective engaging with the intervention. Further, as the “face” of digital health interventions, coaches may serve as the point of contact – or be contacted frequently – regarding usability problems and reports of “bugs and glitches.” One recent study, for example, suggested that almost 20% of questions that clients asked coaches were technical in nature.^22^ Thus, a protocol should consider how the coach will help address or serve as a liaison for technical issues, promote work-arounds for those technical issues, and identify client misunderstandings of program functions that are not actually technical issues. Again, having a thorough understanding of how the intervention is *supposed* to work can help coaches rapidly resolve many technological questions.

### Coaching techniques

In most existing models of coaching, coaches work with clients to facilitate adherence to and understanding of the concepts and exercises presented in the digital mental health intervention. Some coaching happens in the form of setting expectations, normalizing challenges, and helping clients engage in problem-solving. Due to the asynchronous nature of text-based communication, and because the coach is not there to “read” the client’s reaction to messages, the potential for ambiguity is heightened. Using motivational interviewing techniques can be particularly effective.^30^ Nevertheless, when coaching via text, some motivational interviewing techniques appear to be more fruitful than others. We have found eliciting both preparatory and mobilizing change talk to be more helpful, for example, than using reflections, although there remains a need for additional research on how to best use motivational interviewing in the context of text-based coaching with digital mental health interventions.

The increased ambiguity found in text means some motivational interviewing techniques (such as amplified reflections, agreeing with a twist, siding with the negative) may not be appropriate, as clients who misread the tone behind a message may simply not respond and thus render the coaching relationship ineffective. Simple reflections (repeating, rephrasing) can be perceived as annoying or difficult to respond to, as clients are not accustomed to receiving messages that do not move conversations forward incrementally. However, other strategies, featured in Table 1, that are somewhat less subject to interpretation and are more conversational in nature may be particularly useful. These include the use of double-sided reflections, normalizing, emphasizing personal choice, and reflecting feelings.

**Table 1.** Application of recommended motivational interviewing techniques.

Motivational interviewing technique	Sample message
Double-sided reflections	“On the one hand, you’re comfortable in your current situation, and on the other hand, you want to feel better.”
Emphasizing personal choice	“Making these changes is important to you. You’ll do it when you’re ready.”
Reflecting feelings	“You’re feeling confused about what to do next.”
Asking permission	“It sounds like you’re having some trouble making a plan to practice using skills. Can I suggest some strategies that other clients have found helpful?”
Using open-ended questions	“Tell me, what’s been going well in practicing your skills this week?”
Normalizing	“I’ve heard from other clients that it can take some time to see a benefit from applying the skills in this module. Continuing to use the module is a great way to grow your skills!”

Establishing goals and monitoring progress toward goals is central to coaching. While some coaching protocols will include the coach providing feedback on written homework assignments and supporting skill acquisition, other coaching protocols may focus more on program usage. If a coaching protocol defines the role of the coach as primarily supporting engagement with the app or website, then a usage goal may be relatively easy to generate. In some types of interventions, it may take skillful communication to identify and establish a client’s goals. For example, if a coaching protocol defines the role of the coach to include providing support in the acquisition of skills, a coach for a depression intervention may initially hear that a client wants to “feel better” and then collaborate with the client to identify specific measurable goals that are under the client’s control, such as applying for new jobs and spending more time with friends. After goals are established, monitoring progress toward those goals can be incorporated into a coaching protocol with the use of routine check-ins and selected motivational interviewing strategies to address barriers to goal completion.

### Coaching supervision and oversight

In this section, we briefly summarize the roles of supervision and oversight from the perspective of the coaching protocol. In general, during training, we recommend a period of time when all message exchanges are reviewed by a supervisor; the length of this period, however, will vary depending on the complexity of the coaching protocol and the past education and training of the coach. Based on our experiences with digital mental health interventions, we recommend that coaches have weekly individual and/or group supervision following training, which can decrease in frequency as coaches gain experience, especially for lower-complexity coaching. Depending on the nature of the intervention and the experience level of the coaches, coaches should be given a clear definition of the limits of their role. For example, a coach with limited mental health treatment experience may be required to consult with an experienced supervisor when issues arise around the client’s safety risk, symptom deterioration, negative effect of treatment, treatment dissatisfaction, or coaching impasse. Alongside the limits should be information on what to do when a higher level of support is warranted, as stated in the previous section. For example, while a coach may have autonomy to freely communicate with clients in most circumstances, it may be warranted to involve a clinical supervisor in specific instances. Such instances will vary based on the treatment target and population, but could include a supervisor intervening in instances of suicidality or the disclosure of reportable abuse. Thus, a protocol should provide guidance to coaches on what types of situations may warrant involving a clinical supervisor, as well as procedures for how to engage that supervisor.

Once coaching protocols are formulated, developing a rating scale or checklist of expected coaching behaviors has significant potential to aid training and supervision of coaches.

It is helpful, for instance, to develop tools to review both adherence to and quality of coaching. For example, the iCBT Therapist Rating Scale was developed to assess whether weekly therapist emails showed fidelity to specific therapist behaviors.^31^ This scale involves rating each therapist email for adherence (absent/present) and quality (inadequate/competent) on the following behaviors: *Builds Rapport*, *Seeks Feedback*, *Provides Symptom Feedback*, *Provides Psychoeducation*, *Facilitates Understanding*, *Praises Effort*, *Encourages Practice*, *Clarifies Administrative Procedures*, and *Communicates Effectively*. This particular scale has high inter-rater reliability and utility in identifying areas where therapists are not following protocols and can thus be discussed in supervision. While this scale was developed for assessing weekly therapist emails during the course of iCBT, similar checklists could be developed to monitor text-based coaching.

Relatedly, it is beneficial to develop checklists for assessing undesirable coaching behaviors, or behaviors that are viewed as counterproductive to the coaching relationship.^31^ Such checklists are useful during training, but are also beneficial longer term as an efficient method of monitoring coaching quality for problems that could be particularly detrimental for clients. The iCBT Undesirable Therapist Behaviour Scale represents an example of a scale that could be used broadly for coaches of digital mental health interventions, regardless of level of training or type of program. This scale was developed by examining the presence of undesirable therapist behaviors in emails sent to clients in the context of iCBT for depression and anxiety. The following undesirable behaviors were identified as important to monitor in emails sent to clients: *inadequate detail, unaddressed content, unsupportive tone, missed unexplained correspondence, inappropriate self-disclosure*, and *unmanaged risk*.

## Coach protocol

### Structuring communication

Plans for efficiently structuring communication are dependent on both the communication medium (SMS vs email) and on workflow (one outgoing message per week vs multiple). While coaching messages need to be relatively brief, the definition of brief is different for email compared to SMS. A client is much more likely to closely read a five-sentence message sent by email than they are to closely read a similar length message sent by SMS. Thus, if coaching is being delivered primarily through SMS, coaches should aim to limit most messages to 160 characters or less, the standard character limit for a single SMS message. While most modern mobile phones and networks rebuild messages up to 1600 characters, there are several problems with longer messages. Sometimes longer messages can be rebuilt improperly (e.g. segments get delivered out of order), making it harder for the message recipient to understand. But perhaps more importantly, people are used to SMS messages being short; violating those norms may be more likely to result in a negative reaction to the message. Coaches should also be aware of the types of plans people have, as some people may pay per message.

Overall, brevity is valuable regardless of whether communication is via SMS or email formats. Length of messages, however, is also related to how much content is available on the digital mental health interventions. If interventions are content heavy, it is often the case that long emails are perceived as being redundant to content and overwhelming. If interventions, however, have less content, it may be necessary for coaches to have more elaborate messages. In general, people are more likely to read and respond to short messages quickly. In addition to communication medium considerations, the level of brevity is dependent on the coaching workflow. It is not uncommon for a coach to find that there are two, three, or even more issues that would be helpful to address with a client at any given time. If a coach’s workflow includes frequent communications with the client, then rather than attempting to address everything at once, the coach should carefully consider each issue and prioritize one or two per exchange. For example, a coach may wish to 1) remind a client to do something in the intervention, 2) ask the client how they are doing because changes were observed in a weekly assessment, and 3) congratulate the client on their progress in some part of the intervention. For an engaged client who tends to respond to messages, a coach may prioritize sending a congratulatory message because the coach has past evidence that the client will likely respond, and then the coach can address one of the other points. For a less-engaged client who typically takes longer to respond to messages and use intervention techniques, a coach may prioritize bolstering hope or providing tips related to undertaking strategies with the goal of engaging the client in the digital mental health intervention. For coaching workflows that are on a longer cycle, such as weekly contact, then longer messages may be required. In those cases, it is important to organize the communication so that each point is clear and that the totality of the message is not overwhelming. The coaching protocol can provide guidelines to facilitate how coaches should prioritize responding, such as if there are specific types of messages like reminders that should be sent to support intervention delivery.

The more a coach says in a message, the less certain the coach may be about what the client is taking away from the message. Thus, if a coach brings up multiple issues in the same message, the client may not respond to the item that the coach considers highest priority within the message. Additionally, the issues perceived by the client as lower-priority may subsequently “get lost” and not have the same impact. Thus, if a coach’s workflow involves one (or less) outgoing message per week, the messages need to be strategically designed. When a message begins with a question that is followed by some kind of a statement (e.g. a reinforcing comment), clients tend to ignore the opening question altogether, simply because it was placed at the beginning of the text string. For example, consider the following message to a client, in which the goal is to help the client develop a plan to engage with the intervention: “How can you plan to use the intervention this week? I know it can be difficult to remember to log in when your schedule is so busy.” A client may only respond to the validation portion of the message, such as by answering with “Yes, it is tough because I have so many things on my plate lately – I feel very overwhelmed!”, and avoid the prompt to make a plan. Given the client’s response, the coach may be challenged to re-prompt the client again to make a plan. Thus, in both email-delivered and SMS-delivered coaching messages, any questions posed by the coach should be highlighted in some way through formatting (e.g. bolding, italics, indenting, quotes, a list). One approach is to put questions in bold or to place questions together at the end of the message to grab the client’s attention and prompt a response.

### Use of telephone calls

In primarily text-based coaching protocols, telephone calls can be used to support specific goals. While telephone-based coaching comes with some constraints (e.g. potentially higher costs for time spent on telephone, delays in communication due to scheduling difficulties), there are a number of benefits to live synchronous communication, including the ability to further support engagement and to facilitate a more rapid exchange of information. Thus, in developing coaching protocols, researchers should make decision rules regarding if and when telephone calls would be appropriate. Our groups have found that text-based coaching is generally sufficient for interacting with most clients, but we use telephone calls in specific circumstances (e.g. therapists sent around nine emails compared to one phone call during an eight-week transdiagnostic Internet-delivered cognitive behavior therapy intervention^32^; or if clients report a distinct preference for a telephone call). One approach is to use phone calls at the onset of a new intervention in order to build rapport and set expectations of a coaching relationship. We have also provided the option of telephone calls or text-based messages early in the course of some interventions, and then switched to messages as a standard means of communication. We have found it particularly useful to set the expectation with clients that telephone calls may be made when clients have fallen out of contact via messaging, there is miscommunication through messages, or clients appear to be at risk of suicide or are showing significant deterioration in symptoms.

The guidance presented in this paper is also relevant for the development of telephone-based support protocols. Researchers may choose to develop a support protocol delivered primarily by or entirely by telephone calls for a number of reasons, including client preference and technical system limitations. While a thorough discussion of telephone-based coaching is beyond the scope of this paper, we highlight that many of the main themes are transferrable to telephone-based coaching. For example, telephone-based coaching for a digital mental health intervention should remain distinct from telephone-based counseling or therapy. In programs supported by telephone-based coaching, the presentation of psychoeducation and skills should still primarily be provided by the digital tools while the human coach provides support, whereas in telephone-based counseling or therapy, the clinician is typically responsible for introducing knowledge and supporting skill acquisition and the boundaries between information sharing and provision of support are often not apparent. Similarly, the length of a telephone-based coaching session is, in part, dependent on the workflow of the coach. That is, a 30-minute coaching call may be appropriate for infrequent contact, whereas a 5–10-minute call 10-minute call may be more appropriate for protocols with more frequent contact.

### Protocol flexibility

In developing a protocol and training coaches, the degree to which that protocol should be adhered to strictly versus flexibility needs to be considered. There are a number of instances in which flexibility may be considered, including adapting to engage an out-of-contact client, shifting the objective of messaging, or choosing a communication medium that best serves the objective of the communication. For example, a protocol may state that coaches should message clients twice a week, and respond to messages from clients when received. If a client has not responded to their coach in a set amount of time (perhaps two weeks), it may be advisable for the coach to outreach by telephone, and then decrease text messages to that client to once a week for the remaining weeks of the intervention (or until the client has responded again). Similarly, a coach may have already messaged a client twice in one week, and then realize that there is a significant event occurring later in the week that the client may appreciate a supportive message around. It may be advisable that the coach send a third message, and then resume standard use of the protocol in subsequent weeks. In most instances, exchanges with clients regarding technical problems (i.e. usability issues and “bugs and glitches”) should not count towards the number of recommended messages per week in a protocol. It may also be easier for coaches to communicate with clients by telephone to address a technical problem; as such, the protocol should clarify whether and when unplanned telephone communication is appropriate.

Consider as well how weekends and holidays/sick time impact intervention delivery. If a protocol indicates that a client should receive a message at the start of each week in the program, coaches will need guidelines for when to send messages if one of those days occurs on a holiday or if they are sick. For example, a coach may send a message reading “I see next Monday is a holiday. I’ll be messaging you on Tuesday.” In general, there should be a system in place in the event that coaches are unexpectedly absent (e.g. administrative assistant sends brief email alerting clients to the coaches’ absence due to illness). Similarly, if a protocol indicates that coaches should email clients the day after starting the intervention, but the next day falls on a Saturday, guidelines should be specified for this occurrence (or, depending on the clinical importance of the timing of message delivery, avoid starting clients with the intervention on Fridays). A strategy also needs to be in place for when coaches take planned vacation during the intervention (e.g. an alternate coach provides coaching, or coaching is paused).

A coaching protocol may specify that coaching should be offered for a set duration of time (e.g. two months). However, there may be situations in both research and clinical practice settings when the duration of the intervention should be modified. A coach (perhaps in consultation with a supervisor) may decide that a client should be allowed extra time in the intervention based on client-specific circumstances that is necessary to support their overall progress or well-being. For example, it may be clinically indicated to briefly extend the intervention if ending the intervention “on time” would result in a lapse in necessary care before the client transitions to a new care plan with a new care provider, or when there is a crisis near the scheduled end date of the intervention. Conversely, there may be indications that the treatment duration should be shortened. For example, it may be appropriate to monitor for any type of clinical worsening that would indicate that the client be referred to more intensive services before the digital intervention is set to end. If these types of changes in the duration of the intervention are deemed appropriate, it may be helpful to provide guidelines in the protocol to support the determination for changes to the delivery.

## Conclusions

In summary, this paper offers guidance on developing and defining coaching protocols for digital mental health interventions. An overview of these recommendations is provided in Table 2. While we have focused on mental health, many of the recommendations included can be extended to other types of digital health interventions, such as those targeting weight loss or smoking cessation. For coaching support in digital health interventions to be effective and efficient, it must be well-designed. Thus, researchers and practitioners who are tasked with developing coaching protocols should consider the scope of coaching for the intervention, the characteristics of and training of coaches, specific coaching techniques, how to structure communication with clients and also how to train, supervise, and monitor coaches. Taken together, devoting attention to developing the coaching protocol for a digital health intervention will advance the provision of human support in digital health interventions, towards the ultimate goal of establishing engaging and effective interventions.

**Table 2.** Summary of considerations for designing a text-based coaching protocol.

Domain	Recommendation
Overall protocol considerations	Differentiate the goals of coaching from psychotherapy
	Set expectations at the outset regarding response latency
	Delineate message frequency
	Identify criteria for emergency procedures and monitoring
Coach characteristics and training	Consider coaches’ training level
	Consider coaches’ workflows
	Require coaches to have or obtain training in using the intervention
	Consider whether and how coaches will address technological issues
Coaching techniques	If using a coaching model derived from synchronous intervention, adapt for asynchronous communication
	Help clients establish goals and monitor progress toward goals
Content	Be succinct (limit most text messages to ≤160 characters)
	Prioritize message content based on the client and intervention goals
	Place instructions and questions at the end of the message
	Provide templates to help with efficiency
Other	Consider the role of telephone calls to support specific goals
	Delineate ways in which the coaching protocol has flexibility for delivery
Training, supervision and monitoring practice	Offer supervision or oversight, particularly with new coaches
	Develop checklists for assessing adherence to and quality of expected coaching behavior
	Develop checklists for assessing negative coaching behaviors
